# Gut dysbiosis and constipation in patients with end-stage renal disease: pathogenic associations and therapeutic interventions

**DOI:** 10.3389/fcimb.2026.1884822

**Published:** 2026-08-18

**Authors:** Zhiqiang Shen, Lin Gao, Yao Wang, Senxin Li, Lu Lei

**Affiliations:** 1 Department of Hemodialysis, Pujiang County People’s Hospital, Chengdu, Sichuan, China; 2 Department of Spleen and Stomach Diseases, Pujiang County Hospital of Traditional Chinese Medicine, Chengdu, Sichuan, China

**Keywords:** constipation, end-stage renal disease, gut dysbiosis, probiotics, therapeutic intervention

## Abstract

Chronic kidney disease (CKD) is a global, life-threatening condition that can progress to end-stage renal disease (ESRD) without effective medical intervention. Constipation is among the most common gastrointestinal disorders and is closely linked to disease progression and poor outcomes in patients with ESRD. An increasing body of evidence suggests that both CKD patients and those with constipation experience significant changes in gut microbiota composition and face impaired intestinal barrier function. Dysbiosis of the gut microbiota is characterized by a reduced abundance of short-chain fatty acid-producing bacteria and increased levels of proteolytic and urease-positive bacteria, which are associated with elevated intestinal permeability and renal damage. Given the complex interplay between ESRD, constipation, and gut dysbiosis, microbiota-targeted interventions show promise as potential therapeutic strategies for managing constipation in ESRD patients. This review summarizes the observed changes in the gut microbiota among constipated ESRD patients and explores their potential pathogenic mechanisms. Unlike previous reviews that cover the entire spectrum of CKD, this study focuses specifically on the ESRD population and proposes a bidirectional “microbiota–gut–kidney” vicious cycle framework centered on constipation. Additionally, we synthesize current evidence on microbiota-targeted interventions and highlight the translational gaps and safety considerations relevant to their clinical application.

## 1 Introduction

End-stage renal disease (ESRD) is a clinical syndrome characterized by a constellation of symptoms and metabolic disturbances (such as diabetic nephropathy and hypertensive nephropathy) resulting from various chronic kidney diseases (CKD) (Dai and Yuan, 2025). Primary diseases cause structural damage to the glomeruli or a decline in the glomerular filtration rate (GFR), leading to ineffective filtration of metabolic waste in the circulating blood. The latest 2024 KDIGO Clinical Practice Guidelines for the Evaluation and Management of CKD classify CKD into 5 stages on the basis of the GFR. The most accurate methods for estimating the GFR are serum creatinine and cystatin C levels. The diagnostic criterion for stage G5 disease (ESRD) is a persistently low GFR below 15 mL/min/1.73 m^2^ (KDIGO, 2024). According to the 2023 Global Burden of Disease (GBD) study, the age-standardized mortality rate from CKD among adults (≥18 years) globally is 26.5 per 100,000, representing a 6.1% increase since 1990, with CKD being the ninth leading cause of death worldwide (Global Burden of Disease Study 2023, 2025). A national multicenter cross-sectional study from the 2023 China Chronic Disease and Risk Factor Surveillance revealed that approximately 1.8% of the 176,874 participating adults had ESRD (Wang L. et al., 2023). When kidney damage occurs in the early stages, the kidneys compensate by working harder, leading to severe loss of kidney function before the patient feels uncomfortable. In this early phase, inadequate management of kidney disease risk factors (such as hypertension and diabetes) fails to intervene with the kidneys on time; thus, the golden time window for intervention is missed (Kanbay et al., 2024; Kushner et al., 2024; Cunillera-Puértolas et al., 2025). Renal replacement therapy represents the main therapeutic approach for treating ESRD and includes kidney transplantation, hemodialysis (HD), and peritoneal dialysis (PD). Nevertheless, constrained by the high expense of transplantation and the scarcity of eligible donors, HD and PD remain the primary treatment modalities for ESRD (Hong et al., 2023; Dai and Yuan, 2025).

Gastrointestinal symptoms affect 77%-79% of ESRD patients, among which constipation is the most prevalent disorder and is characterized by difficult defecation and reduced bowel movement frequency (Cano et al., 2007; Camilleri et al., 2017; Khan et al., 2025). A large-scale health check-up cohort analysis in Japan revealed that 9.8% of 1,195,166 participants experienced constipation, with a higher prevalence of constipation as the GFR decreased; the incidence rate of constipation in ESRD patients was 50.7% (Hirano et al., 2025). A Chinese cross-sectional observational study revealed that the constipation occurrence rate in PD recipients was 52.7%, and it was as high as 77.4% in HD patients (Zhang et al., 2022). The etiology of constipation in CKD/ESRD patients is multifactorial: commonly prescribed medications (iron supplements, antihypertensives, potassium binders, phosphate binders, diuretics), metabolic comorbidities (diabetes, hypercalcemia), dietary fiber and fluid restrictions, electrolyte disturbances, uremic toxin accumulation, and dialysis-related effects collectively contribute to its high incidence (Cha et al., 2023). In addition to being a gastrointestinal symptom, constipation is associated with accelerated renal function decline, an increased risk of ESRD progression, increased mortality, and impaired quality of life (Sumida et al., 2017; Park et al., 2025). Traditional management [osmotic and stimulant laxatives and dietary adjustment (Cha et al., 2023)] has limited efficacy in ESRD patients and is associated with safety risks, including volume depletion, electrolyte imbalance, and potential nephrotoxicity. There is an urgent clinical need for a safe and effective targeted therapeutic strategy, with the gut microbiota emerging as a promising research direction.

In healthy individuals, the gut microbiota maintains a mutualistic relationship with the host. These intestinal microorganisms play key roles in assisting host metabolism, maintaining intestinal barrier function, promoting immune system development, and regulating intestinal function through the release of microbial metabolites or fermentation end products. Major metabolites include secondary bile acids (BAs), short-chain fatty acids (SCFAs), tryptophan metabolites, trimethylamine N-oxide (TMAO), and aromatic amino acid metabolites [such as indoxyl sulfate (IS), phenylacetylglutamine (PAG), and p-cresol sulfate (PCS)] (Wang J. et al., 2023). The gut microbiota is a key regulator of intestinal motility, barrier function, and immune homeostasis, and its crosstalk with the kidney is defined as the “gut–kidney axis” (Wong et al., 2014; Rusu et al., 2026). Dysbiosis of the gut microbiota is also a risk factor for chronic constipation (Zhuang et al., 2019). The abundance of the gut microbiota that produces SCFAs is reduced in constipated patients, leading to a significant decrease in SCFA production, which may result in impaired intestinal motility and intestinal barrier function. The changes in the gut microbiota observed in constipated patients are similar to those observed in ESRD/CKD patients (Ikee et al., 2020). Gut dysbiosis may serve as a key bridge connecting the gut–kidney axis, and constipation can further accelerate the decline in renal function in patients with ESRD, creating a vicious cycle.

This review focuses on the potential mechanisms by which the gut microbiota contributes to the development and progression of constipation in patients with ESRD. This study aims to clarify the regulatory role of gut microbiota dysbiosis in ESRD and its pathogenic association with constipation, providing a new pathological and physiological perspective for clinical practice. Current reviews on the gut–kidney axis predominantly focus on CKD stages, with fewer incorporating constipation as a key regulatory node in disease progression. This review specifically focuses on ESRD and dialysis populations rather than all CKD stages and systematically summarizes the gut microbiota characteristics of constipated ESRD patients. Furthermore, it integrates gastrointestinal motility dysfunction, intestinal barrier damage, and toxin amplification pathways to construct a bidirectional “microbiota–gut–kidney” vicious cycle framework centered on constipation. This study aims to shift the management of constipation in end-stage renal disease patients from symptom relief to mechanism-based intervention, providing a theoretical foundation for improving patient quality of life and prognosis.

## 2 Methods

This article is a narrative review. Studies related to gut dysbiosis, constipation, and ESRD were identified through a comprehensive literature search.

### 2.1 Literature search

A literature search was conducted in English databases, including PubMed and Web of Science. The search scope covered literature published from January 1, 2015, to April 30, 2026.

### 2.2 Search terms

The following search terms were used to identify the literature: (End-Stage Renal Disease OR End-Stage Kidney Disease OR Hemodialysis OR Uremia OR Chronic Renal Failure) AND (Constipation OR Low Bowel Frequency OR Bowel Dysfunction OR Gut Dysbiosis) AND (Gut Dysbiosis OR Gut Microbiome).

### 2.3 Literature screening criteria

All types of study designs (randomized controlled trials, cohort studies, case–control studies, cross-sectional studies, etc.), as well as systematic reviews, meta-analyses, mechanistic studies, and preclinical experimental studies, were included. Two researchers independently screened abstracts of the literature and reviewed all potentially eligible retrospective studies. Any discrepancies were resolved through discussion, and a third reviewer made the final decision if necessary. Manual backward citation tracking based on the references of the included studies was performed to supplement other relevant studies.

## 3 Characteristics and clinical correlations of alterations in the gut microbiota and their metabolites in ESRD patients

### 3.1 Characteristics of the gut microbiota and metabolites in healthy individuals

In healthy adults, the gut microbiota is dominated by obligate and facultative anaerobes, with Firmicutes (median 49.5–59.6%) and Bacteroidetes (28.0–33.4%) as the two dominant phyla, followed by Proteobacteria (3.4–5.9%), Actinobacteria (2.3–3.7%) and Verrucomicrobia (0.5–1.0%) at relatively low abundances (Glazunova et al., 2025). A healthy gut microbiome is characterized by high microbial diversity and compositional stability (Lozupone et al., 2012). *Bacteroidetes* and *Firmicutes* are the main producers of SCFAs. TMAO is generated by hepatic oxidation of trimethylamine (TMA), which is a precursor produced by intestinal bacteria from dietary choline and L-carnitine (Chambers et al., 2018; Singh et al., 2022). According to a Chinese clinical study, the fecal SCFA concentration in 30 healthy adults (mean age 51.93 ± 8.62 years) was 58.93 µmol/g (range: 40.35–76.18 µmol/g) (Zhong et al., 2021). SCFA levels in healthy individuals vary across individuals and are influenced by multiple factors, including diet, lifestyle, and age. In a cohort of 157 healthy Thai volunteers with a mean age of 23 years (age range: 18–59 years), fecal and plasma samples showed total SCFA concentrations of 34.07 ± 15.26 µmol/g and 60.01 ± 45.87 µM, respectively. Among the total SCFAs in the feces, 12.61 ± 6.45 µmol/g of acetic acid, 9.61 ± 5.09 µmol/g of propionic acid, and 7.27 ± 4.42 µmol/g of butyric acid (Manokasemsan et al., 2024).

At the metabolic level, SCFAs (mainly acetate, propionate and butyrate) are the core beneficial metabolites of gut microbial fermentation, while gut-derived UTs such as IS, PCS, PAG and TMAO are maintained at low circulating levels under physiological conditions (Tang et al., 2013). In 11 healthy American volunteers without dietary restrictions (aged 39 ± 11 years), urinary PCS levels were 100 (47–124) mg/d/1.73 m², and IS levels were 47 (40–59) mg/d/1.73 m^2^ (Patel et al., 2012). A Chinese multicenter study revealed that the plasma TMAO concentration in 649 healthy adults (aged 18–85 years) was 1.70 (1.09–2.53) µM (Fang et al., 2025). The German study included 694 participants who were free of clinical cardiovascular disease, chronic kidney disease, and type 2 diabetes (54% male; age range: 25–82 years) and revealed significant sex disparities in circulating TMAO levels. In male subjects, the median plasma TMAO concentration was 3.91 (2.87–6.10) μmol/L, with a reference range of 1.28–19.67 μmol/L. In female subjects, the median plasma TMAO concentration was 3.56 (2.41–5.15) μmol/L, with a reference range of 1.08–17.12 μmol/L (Gessner et al., 2020). In a U.S. study of 16 volunteers with no history of kidney disease, the PAG concentration was 0.05 ± 0.02 mg/dl (Sirich et al., 2014). Owing to factors such as dietary variations and significant individual differences, there is no universally accepted reference range for SCFAs and UTs; current values primarily serve as research reference ranges.

The stable production of short-chain fatty acids and low levels of UTs maintain normal intestinal peristalsis, barrier function, and metabolic balance.

### 3.2 Changes in the gut microbiota and metabolites in ESRD patients

ESRD-associated gut dysbiosis is broadly characterized by reduced microbial α diversity, depletion of SCFA-producing taxa, and overgrowth of proteolytic and urease-positive bacteria, accompanied by insufficient SCFA synthesis and excessive accumulation of gut-derived UTs (Sirich et al., 2017; Zhang et al., 2024) (**Table 1**).

**Table 1:** Characteristics of the gut microbiota and its metabolites in ESRD patients.

Control group	Experimental group	Gut microbiota abundance		Metabolic products		References
		Increased	decreased	Healthy	ESRD	
Healthy population	HD patients	CAG-352, *Neisseria*, *Akkermansia*, Lachnospiraceae_unclassified	*Bilophila*, *Haemophilus*, *Coprococcus*, *Citrobacter*, *Lactiplantibacillus*, *Alysiella*, *Leuconostoc*, *Selenomonas*, Erysipelotrichaceae_UCG-003, *Lacticaseibacillus*, *Enterobacter*, *Coprobacter* and *Neobitarella*	NA	NA	(Zhang et al., 2024)
Healthy control group	HD patients	*Brachybacterium*, Enterobacteriaceae, *Catenibacterium*, Moraxellaceae, Polyangiaceae, Halomonadaceae, *Thiothrix*, and *Nesterenkonia*	Sutterellaceae, Bacteroidaceae, and Lactobacillaceae families	NA	NA	(Vaziri et al., 2013)
Healthy control group	HD patients	Enterobacteriaceae and Enterococci	*Bifidobacterium*	NA	NA	(Hida et al., 1996)
Healthy control group	ESRD patients	*Eggerthella lenta*, *Flavonifractor* spp (mainly *F. plautii*), *Alistipes* spp (mainly *A. finegoldii* and *A. shahii*), *Ruminococcus* spp and *Fusobacterium* spp	*Prevotella* spp (mainly *P. copri*), *Clostridium* spp and *Roseburia* spp, *Faecalibacterium prausnitzii* and *Eubacterium rectale*	NA	NA	(Wang et al., 2020)
Healthy volunteers	ESRD patients	Oscillospiraceae (such as *Oscillibacter*, *Flavonifractor*), Lachnospiraceae (such as *Blautia*, *Faecalimonas nexilis*, *Hungatella effluvii*), Eggerthella	*Roseburia*, *Faecalibacterium*, *Coprococcus*	IS (0.34 ± 0.28), PCS (0.7 ± 1.06), PAG (0.41 ± 0.16), TMAO (0.19 ± 0.27)	IS (36.42 ± 17.17), PCS (29.86 ± 21.63), PAG (37.47 ± 25.31), TMAO (9.78 ± 8.65)	(Zhang et al., 2023)
Normal renal function	ESRD patients	Christenellaceae_R_7_group (Christensenellaceae family), Clostridium innocuum group (Erysipelotrichaceae family)	*Megamonas*, *Fusicatenibacter*, *Agathobacter*	FA 0.60 (0.34, 1.33), AA 64.47 (53.52, 87.59), PA 19.19 (14.43, 28.23), BA 12.3 (6.75, 19.14), VA 1.9.(0.33, 2.63); IS 0.5 (0.5, 1.1), PCS 1.2 (0.5, 4.1), PS 0.3 (0.3, 0.7)	FA 0.4 (0.12, 0.66), AA 50.76 (31.63, 64.91), PA 12.33 (8.97, 18.86), BA 7.77 (4.76, 11.53), VA 1.52 (0.33, 2.28); IS 23.1 (16.3, 27.7), PCS 22.7 (13.5, 30.3), PS 7.8 (3.8, 15.3)	(Koshida et al., 2023)
Healthy controls	PD patients	*Fusobacterium*, *Clostridium*, and *Eucalyptus* bacteria	Bifidobacteria, *Faecalibacterium*, *Subdoligranulum*, *Roseburia*, Erysipelotrichaceae, Lachnospiraceae, and Clostridiales	IS (0.35 ± 0.26), PCS (0.63 ± 0.81), TMAO (0.19 ± 0.28)	IS (27.00 ± 14.81), PCS (20.68 ± 15.54), TMAO (5.14 ± 3.45))	(James et al., 2025)
Healthy controls	PD patients	*Blautia*, Escherichia-Shigella, *Treponema succinifaciens*, *Pseudomonas*, unclassified Lachnospiraceae, *Clostridium* in the Erysipelotrichaceae family, and *Eisenbergiella*	*Propionibacterium*, *Roseburia*, *Subdoligranulum*, *Fusobacterium*, *Lachnospira*, *Ruminococcus*, Eubacterium eligens group, Lachnospiraceae NK4A136 group, and *Sutterella*	IS 0.51 (0.28-0.70), PCS 0.61 (0.11-1.29)	IS 30.93 (24.32-32.06), 8.53 (1.91-14.83)	(Lin et al., 2022)

This table summarizes the gut microbiota abundance and metabolic product levels in healthy control groups versus patients with hemodialysis (HD), end-stage renal disease (ESRD), or peritoneal dialysis (PD) across multiple studies. “NA” indicates that there are no data or that the data are unavailable in the original reference literature. The concentration units for uremic toxins in feces (such as IS, PCS, PAG, PS, and TMAO) are μg/mL; the concentration units for SCFAs are µmol/g; and the concentration units for serum uremic toxins are mg/L. AA, acetic acid; BA, butyric acid; IS, indoxyl sulfate; PA, propionic acid; PAG, phenylacetylglutamine; PCS, p-cresyl sulfate; SCFA, short-chain fatty acid; TMAO, trimethylamine N-oxide; VA, valeric acid.

Multiple independent cohorts have consistently shown that compared with healthy controls, HD patients have reduced gut microbial species richness and diversity. At the taxonomic level, HD patients exhibit a consistent decrease in SCFA-producing bacteria (Hida et al., 1996; Vaziri et al., 2013; Rysz et al., 2021). Moreover, the abundance of aerobic and UT-associated bacteria, including Enterobacteriaceae, enterococci, *Eggerthella lenta*, and *Fusobacterium nucleatum*, and taxa such as *Brachybacterium*, *Catenibacterium*, and Oscillospiraceae, significantly increased. A large metagenomic study further confirmed the enrichment of Oscillospiraceae (e.g., *Oscillibacter* and *Flavonifractor*), Lachnospiraceae (e.g., *Blautia* and *Hungatella effluvii*) and *Eggerthella* in HD patients (Zhang et al., 2023). At the metabolic level, compared with healthy controls, HD patients consistently have reduced fecal SCFA concentrations (including formate, acetate, propionate and butyrate) and elevated fecal pH, along with significantly higher serum levels of IS, PCS, PAG and TMAO (P < 0.01) (Moco et al., 2012; Wang et al., 2020; Koshida et al., 2023; Zhang et al., 2023). Correlation analyses have revealed that taxa such as *Megamonas* and Christensenellaceae_R_7_group are strongly associated with the accumulation of circulating IS and PCS (Koshida et al., 2023).

Patients with PD exhibit an overall dysbiosis pattern similar to that of HD patients, characterized by depleted SCFA-producing bacteria and elevated circulating UTs. Specifically, PD patients show a reduced abundance of SCFA-producing taxa, including *Bifidobacterium*, *Faecalibacterium*, *Subdoligranulum*, and *Roseburia*, along with decreased fecal SCFA concentrations (Bao et al., 2022). Concurrently, UT-associated bacteria such as *Fusobacterium*, *Clostridium*, and *Akkermansia* are enriched, and serum levels of IS, PCS, and TMAO are significantly increased (Hoibian et al., 2018). Additional PD-specific taxonomic changes include increased abundance of *Blautia*, *Escherichia*-*Shigella*, *Pseudomonas*, and *Eisenbergiella* and decreased abundance of *Propionibacterium*, *Ruminococcus*, and *Sutterella* (James et al., 2025). Notably, Australian researchers stratified 43 PD patients by circulating UT levels and reported that patients in the high-UT group had a significantly lower residual glomerular filtration rate (GFR) (5.89 ± 2.22 vs. 8.04 ± 2.44 mL/min/1.73 m², P < 0.01), supporting a close link between gut microbial dysbiosis and renal function deterioration (Lin et al., 2022).

These findings indicate that the primary alterations in the gut microbiota of patients with ESRD are characterized by variably reduced diversity and significant shifts in microbial abundance. The observed gut dysbiosis is closely associated with decreased levels of SCFAs, elevated concentrations of UTs, and impaired renal function. However, it is important to emphasize that the current evidence supports an association rather than a causal relationship between gut microbial imbalance and the progression of ESRD. Furthermore, the microbial signatures identified across studies show only partial consistency. This heterogeneity may have arisen from multiple factors, including differences in study populations (e.g., HD vs. PD, geographic regions, and baseline comorbidities), variations in 16S rRNA sequencing targets and bioinformatics pipelines, and insufficient adjustment for dietary intake and concomitant medications. These methodological and clinical diversities underscore the need for standardized protocols and well-controlled longitudinal studies to strengthen the interpretability and reproducibility of future findings.

## 4 Mechanisms through which the gut microbiota and constipation influence the progression of CKD-ESRD

**Figure 1:**
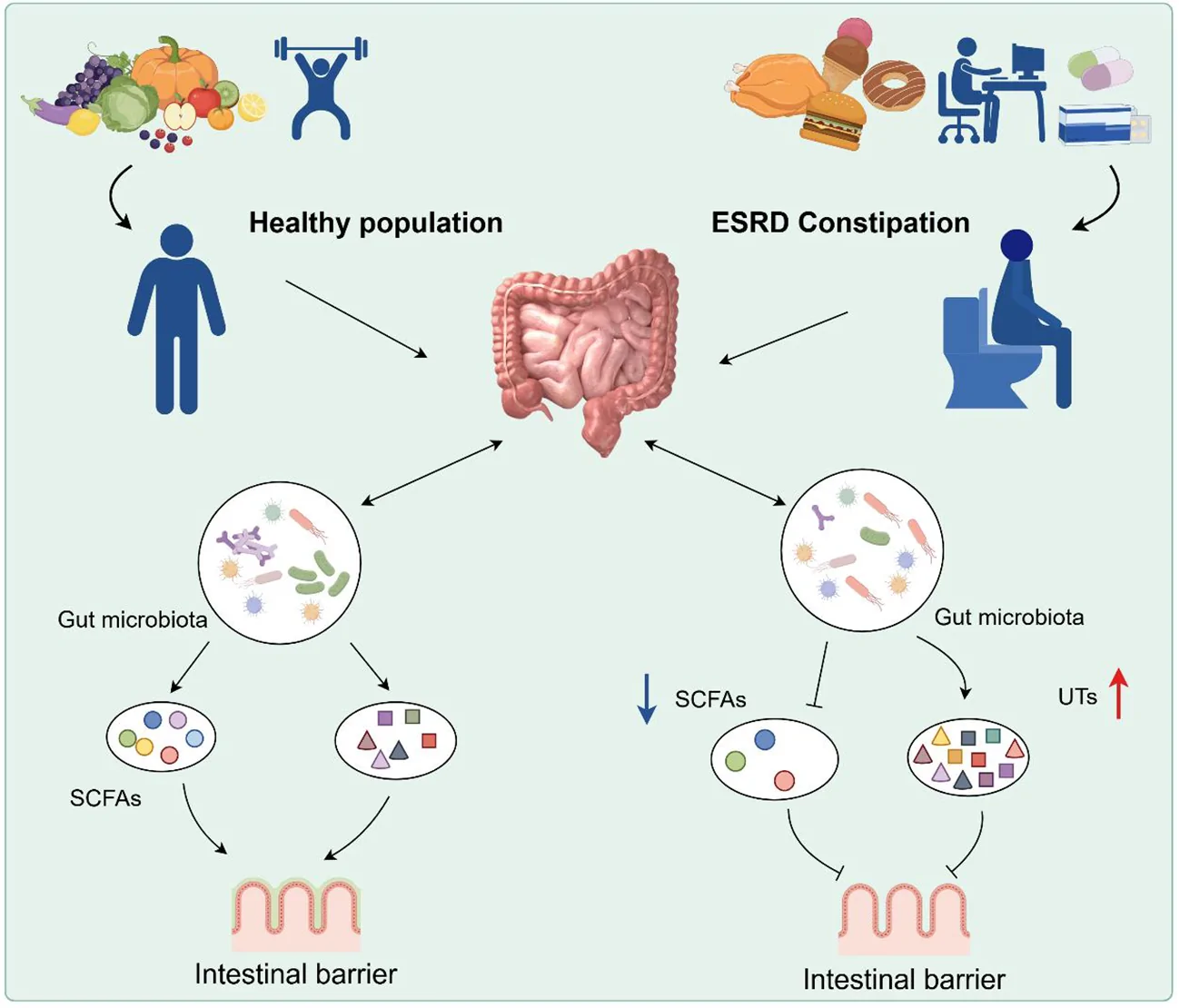
Correlations between the gut microbiota and constipation. In healthy individuals, the gut microbiota maintains a mutualistic relationship with the host. In patients with ESRD complicated by constipation, there is a decrease in gut microbial diversity, reduced abundance of bacteria that produce SCFA, and increased abundance of UTs-producing bacteria. This results in reduced synthesis of SCFA, elevated levels of UTs, disruption of gut homeostasis, and exacerbation of ESRD. By Figdraw.

The gut microbiota serves as a core mediator of the “gut–kidney” axis. In patients with CKD complicated by constipation, dysbiosis-driven metabolic abnormalities impair intestinal function and accelerate renal deterioration concurrently, thereby forming a bidirectional pathological circuit (**Figure 1**).

### 4.1 Gastrointestinal motility disorders

Gastrointestinal motility dysfunction (slow transit constipation, STC) is the primary manifestation of constipation in CKD-ESRD patients.

#### 4.1.1 Reduced SCFA synthesis impairs colonic motility

Reduced synthesis of SCFAs and impaired colonic motility are observed. Mancabelli et al. analyzed the gut microbiota composition of constipated patients using 16S rRNA sequencing and whole-genome sequencing (Mancabelli et al., 2017). The data revealed a significant reduction in SCFA-producing bacteria (e.g., *Bacteroidetes*) in constipated patients, leading to weakened SCFA-mediated intestinal peristalsis. The neurotransmitter 5-HT is a key factor driving intestinal peristaltic reflexes (Heredia et al., 2013). SCFAs can stimulate the expression of GLP-1 and PYY, thereby regulating 5-HT release and affecting colonic motility. Additionally, butyrate enhances choline acetyltransferase (ChAT) activity in an MCT2-dependent manner, improving colonic transit (Soret et al., 2010). In CKD/ESRD patients, the reduced abundance and synthesis of SCFA-producing microbes impair the supportive role of SCFAs in intestinal function.

#### 4.1.2 Uremic toxin accumulation inhibits intestinal smooth muscle contraction

The accumulation of UTs inhibits intestinal smooth muscle cell contraction. Measurement of UTs levels in hemodialysis patients who had undergone total colectomy revealed extremely low levels, indicating that a large portion of UTs are produced by the colonic microbiota through protein hydrolysis and fermentation (Aronov et al., 2011). Animal experiments confirmed that gastrointestinal transit time in CKD mice was 1.8-fold longer than that in normal mice, with prolonged colonic transit and low fecal water content. Concurrently, the maximum contractile force of colon segments cultured with plasma from hemodialysis patients was significantly lower than that of controls, similar to results from colon segments cocultured with two typical uremic toxins (IS and PCS) (Hoibian et al., 2018). The accumulation of UTs impairs colonic motility. Reduced intestinal motility, by increasing the retention time of the colonic content, promotes the utilization of fermentable amino acids and the generation of UTs, resulting in a vicious cycle (Ramos et al., 2020).

### 4.2 Intestinal barrier damage

Intestinal epithelial integrity is the core defense against pathogenic microbes and their metabolites and is maintained by two complementary protective layers. The first line of defense is a bilayer mucus structure composed of mucin 2 (MUC2) secreted by goblet cells (Okumura and Takeda, 2018). The second is the intestinal epithelial cell layer sealed by tight junction protein complexes, including claudin-1, occludin, and zonula occludens-1 (ZO-1) (Morrison and Preston, 2016).

#### 4.2.1 Gut dysbiosis disrupts barrier structure

In CKD/ESRD, gut dysbiosis disrupts intestinal homeostasis via two interconnected mechanisms. First, urease-positive bacteria decompose urea into corrosive compounds such as ammonium hydroxide (NH_4_OH), which directly degrades tight junction proteins and damages epithelial barrier integrity (Lau et al., 2015; Vaziri et al., 2016). Second, dietary fiber restriction in CKD patients reduces the supply of fermentable substrates to the gut microbiota, leading to the expansion of mucus-degrading bacteria that excessively erode the mucus layer, reduce its thickness, and impair its defensive function (Okumura and Takeda, 2018). Increased intestinal barrier permeability allows uremic toxins and bacterial products such as lipopolysaccharide (LPS) to translocate into the systemic circulation, triggering systemic low-grade inflammation and damaging target organs, including the kidneys, thereby accelerating ESRD progression.

Furthermore, gut dysbiosis disturbs the balance between immune activation and tolerance, inducing abnormal proliferation and differentiation of B and T lymphocytes, promoting autoantibody and proinflammatory factor production, and indirectly driving CKD progression (Honda and Littman, 2016; Thaiss et al., 2016).

#### 4.2.2 SCFAs maintain barrier integrity and immune homeostasis

SCFAs protect the intestinal epithelial barrier through both mechanical and immunological mechanisms. Mechanically, SCFAs are the primary energy source for colonic epithelial cells, and they upregulate the expression of tight junction proteins (claudin-1, occludin, and ZO-1) to enhance barrier integrity (Wang et al., 2012; Meijers et al., 2018). Immunologically, SCFAs inhibit histone deacetylases (HDACs) and activate G protein-coupled receptors (GPR43, GPR41, and GPR109A), thereby suppressing NF-κB signaling and reducing the release of proinflammatory cytokines such as IL-6 and TNF-α (Singh et al., 2014; Vaziri et al., 2016). Butyrate has been shown to inhibit the release of multiple proinflammatory cytokines in colon biopsies from Crohn’s disease patients (Segain et al., 2000; Chang et al., 2014). Additionally, butyrate promotes colonic regulatory T-cell (Treg) differentiation via increased histone H3 acetylation at the Foxp3 gene promoter and conserved noncoding sequence regions, maintaining local immune tolerance and reducing inflammatory damage to the intestinal barrier; moreover, the colonic SCFA concentration is positively correlated with the abundance of colonic Tregs (Furusawa et al., 2013).

### 4.3 Gut–kidney axis toxin amplification cycle

Constipation reduces intestinal motility and prolongs colonic transit time, which increases the absorption of gut-derived uremic toxins into the systemic circulation (Pereira et al., 2020).

Elevated circulating TMAO levels are associated with upregulated SMAD3 and NLRP3 mRNA expression and increased secretion of proinflammatory factors such as TGF-β1 and IL-1β in CKD patients. TMAO may promote renal fibrosis via activation of the TGF-β/SMAD signaling pathway and the NLRP3 inflammasome (El-Deeb et al., 2019). Animal experiments have confirmed that pharmacologically inhibiting TMAO production alleviates tubular fibrosis and improves renal dysfunction in mice with high-choline diet-induced CKD (Gupta et al., 2020).

IS exerts profibrotic effects via the organic anion transporter (OAT)/NADPH oxidase/ROS axis. Nakano et al. demonstrated that IS activates mTORC1 through this pathway, inducing macrophage inflammation and tubular epithelial–mesenchymal transition, thereby promoting renal fibrosis (Nakano et al., 2021). The renal drug transporter OAT1 is also closely involved in the handling of gut-derived metabolites, including p-aminophenol and tryptophan derivatives (Ermakov et al., 2023).

These studies suggest that CKD combined with constipation leads to gut microbiota dysbiosis, reduced intestinal motility, and intestinal barrier damage. The accumulation of UTs promotes renal fibrosis, further decreases the GFR, and reduces the excretion capacity of UTs, thereby accelerating the progression to ESRD.

**Figure 2:**
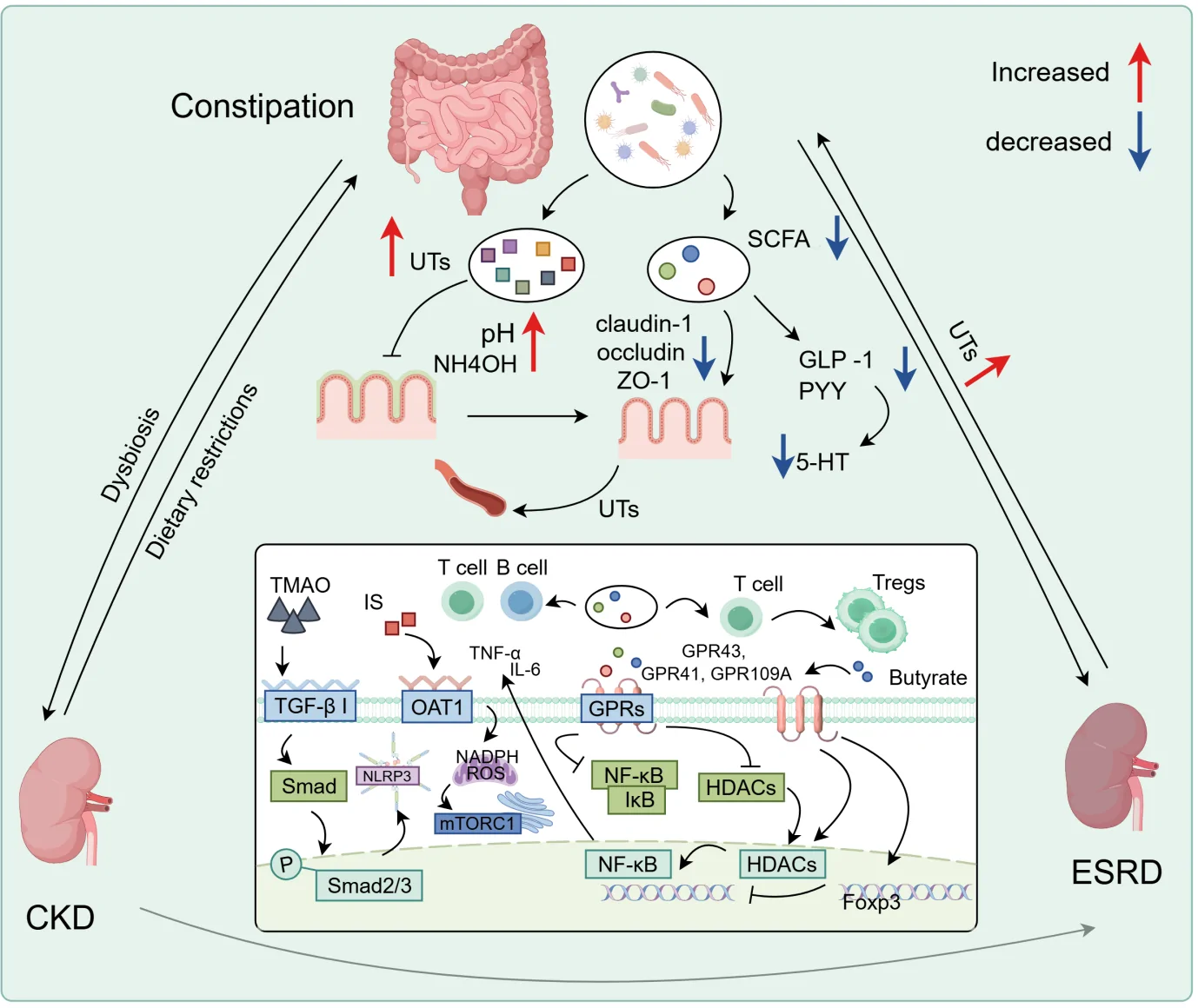
Mechanisms of gut microbiota and constipation in influencing the progression of CKD/ESRD. The gut microbiota is an indispensable component of the “gut–kidney” axis. In patients with CKD complicated by constipation, the abundance and composition of the gut microbiota are abnormal, leading to the dysregulation of microbial metabolites. This, in turn, causes intestinal barrier damage, promotes CKD progression, and ultimately results in ESRD. Constipation can accelerate CKD progression. On the other hand, dietary restrictions, declining renal function, and drug interventions in CKD patients induce gut microbiota dysbiosis. The accumulation of UTs in circulation exacerbates renal injury. By Figdraw.

Collectively, the mechanistic links between gut dysbiosis, constipation, and ESRD progression are biologically plausible and supported by preclinical evidence, but several caveats apply. First, most human data are associative and cannot confirm directionality; whether constipation and dysbiosis are causal drivers or merely epiphenomena of advanced renal disease remains unresolved. Second, many mechanistic inferences are extrapolated from non-CKD animal models or *in vitro* systems, and their relevance to the complex uremic milieu of human ESRD requires validation. Third, the relative contribution of constipation itself versus shared confounders (e.g., dietary fiber restriction, polypharmacy, and physical inactivity) to microbial and metabolic alterations has not been quantified (**Figure 2**).

## 5 Targeted gut microbiota therapeutic strategies for ESRD patients with constipation

**Figure 3:**
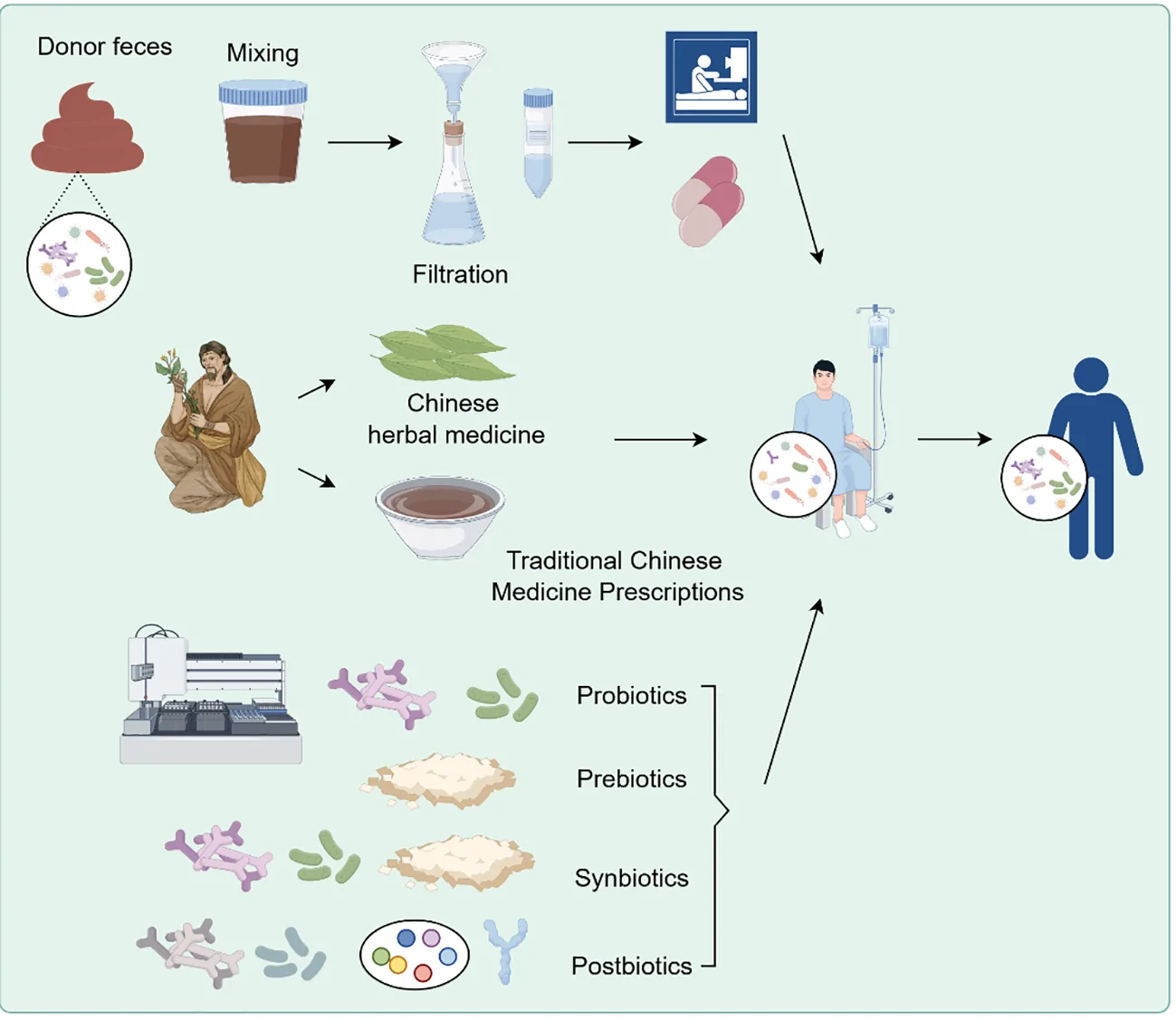
Targeted intestinal microbiota therapy for constipation in ESRD patients. Three strategies for targeted gut microbiota therapy in CKD patients with constipation include FMT, TCM intervention, and microbial agent intervention. By targeting the gut microbiota to reshape gut microbiota homeostasis, these strategies simultaneously improve intestinal function and delay the progression of ESRD. By Figdraw.

Traditional constipation management approaches (such as laxatives and dietary adjustments) have limited efficacy in patients with ESRD and carry risks of electrolyte disturbances and volume depletion, thus necessitating safer, mechanism-based interventions (Xing and Soffer, 2001; Dhondup and Qian, 2017). Growing evidence suggests that targeting the gut microbiota can modulate microbial composition, improve intestinal motility and barrier function, and reduce the production of uremic toxins, making it a promising therapeutic avenue. In recent years, treatment strategies targeting the gut microbiota have included FMT, traditional Chinese medicine and herbal decoctions, probiotics, and prebiotics (Ramezani et al., 2016; Rossi et al., 2016; Zhang et al., 2018; Wang et al., 2024) (**Figure 3**) (**Table 2**).

**Table 2:** Gut-targeted intervention studies in patients with CKD and preclinical animal models.

Treatment	Study population	Dialysis status	Study design	Sample size	Intervention	Constipation endpoint	Microbiome/metabolite endpoint	Renal outcome	Reference
FMT	Stage 2–4 CKD patients with diabetes/hypertension	Non-dialysis CKD	RCT	28	FMT	NA	The abundance of FMT group thick-walled bacteria and *Actinobacteria* decreased, while the abundance of *Bacteroidetes*, *Proteobacteria*, and *Roseburia* increased.	Exhibited more stable renal function parameters (serum creatinine, blood urea nitrogen) and a slower decline in eGFR; 53.8% of patients in the placebo group progressed to more severe CKD, compared to only 13.3% in the FMT group.	(Arteaga-Muller et al., 2024)
	CKD patients (stages 1–5, non-dialysis)	Non-dialysis CKD	Observational single-arm trial	22	FMT	Improvement of gastrointestinal symptoms in 72.7% of patients	NA	Decreased 24h urinary protein; decreased serum creatinine; increased eGFR	(Guo et al., 2025)
TCM	CKD patients with constipation	Non-dialysis CKD	RCT	66	CFS	Significantly improved constipation symptoms; superior to lactulose	NA	Decrease BUN and Scr level.	(Jahanian et al., 2025)
	PD patients with constipation	PD	Single-center, prospective, single-arm observational study	29	TXD	Decreased bloating scores, complete disappearance of hard stools, and a 2.6-fold increase in the incidence of soft stools, indicating significant improvement in constipation symptoms.	Increased gut microbial diversity; enriched *Paraprevotella clara*, *Phascolarctobacterium succinatutens*, *Prevotella copri*	NA	(Peng et al., 2023)
	Maintenance HD patients with uremic pruritus	HD	RCT	139	Bushen Qudu Formula	Significantly improved constipation scores	NA	Reduced serum BUN, CRP and potassium levels	(Vaziri et al., 2014)
Probiotics	Non-dialysis CKD patients (stage 3–5)	Non-dialysis CKD	RCT	75	*Lacticaseibacillus rhamnosus* L34	NA	Reduced free IS and p-cresol sulfate; downregulate intestinal permeability markers such as β-D-glucan; decrease *Bacteroidetes* and enrich *Actinobacteria* to improve gut microbiota dysbiosis;	After intervention, Scr, eGFR, BUN, and urinary protein showed no significant changes	(Leelahavanichkul et al., 2025)
	Type 2 diabetes mellitus complicated by chronic kidney disease and constipation	Non-dialysis CKD	Multicenter, open-label, single-arm study	30	*Bifidobacterium bifidum* G9-1	Improvement in Bristol score and defecation frequency, with marked alleviation of gastrointestinal symptoms such as bloating, incomplete evacuation, and abdominal pain.	A decrease in sulfur-reducing bacteria, iron-reducing bacteria phylum, *Lachnoclostridium*, and *Lachnospira* was observed. Only TMAO toxin showed a significant reduction after 6 weeks; indole sulfate, p-cresol sulfate, and fecal short-chain fatty acids remained unchanged.	The eGFR, Scr, and HbA1c showed no significant changes; only a marked decrease in urinary albumin was observed at week 6, with no sustained benefit at 12 weeks.	(Ikeda et al., 2026)
Prebiotics	Maintenance HD patients with constipation	HD	Single-arm interventional study	15	Partially hydrolyzed guar gum	The CAS constipation score decreased from 5.1 to 3.0, there was a significant improvement in Bristol stool form, and abdominal symptoms such as bloating and incomplete evacuation were alleviated.	The abundance of *Bifidobacterium*, *Bacteroidetes*, and *Lactobacillales* was upregulated, while *Clostridium putrificum* was reduced; fecal total SCFA increased by 1.58 times, with significant elevations in acetic acid, propionic acid, and butyric acid.	Scr, BUN, serum calcium and phosphorus, and albumin showed no significant changes.	(Miyoshi et al., 2020)
Synbiotics	Maintenance HD patients with constipation	HD	RCT	60	Compound synbiotic (Lactobacillus, Bifidobacterium, fructooligosaccharides)	Significantly improved constipation symptoms; improved quality of life	IS did not show a significant decrease after intervention	NA	(Lydia et al., 2022)
	Stage 3–5 CKD patients	Non-dialysis CKD	Single-blind randomized placebo pre-trial	23	Synbiotics (Lactobacilli/Bifidobacteria + FOS/Inulin)	The GSRS scale showed significant improvement in hard stools, abdominal pain, and constipation total scores	Free IS is significantly reduced, while total IS and PCS show a downward trend only.	No significant intergroup differences were observed for eGFR, Scr, BUN, CRP, and IL-6; urea nitrogen and calcium-phosphorus product showed mild decreases during the washout period.	(Cosola et al., 2021)
Postbiotics	Adenine-induced CKD male mice	Preclinical	Animal experiment	60	Inactivated *Lactiplantibacillus plantarum* MA2	NA	Reduced IS, PCS, and TMAO within the body; enhance gut microbiota diversity, enrich *Lactobacillus*, and downregulate *Proteobacteria* and indole-producing pathogenic bacteria	Upregulation of claudin-1 repairs the intestinal barrier, reducing renal LPS, TLR4, and IL-1β; significantly lowers serum creatinine, urea, and uric acid; alleviates renal fibrosis and tubular apoptosis, and enhances renal antioxidant capacity.	(Geng et al., 2025)
	Cisplatin-induced CKD in male mice	Preclinical	Animal experiment	20	Inactivated *Lactiplantibacillus plantarum* MA2	NA	NA	No significant differences were observed among groups for Scr, BUN, CRP, and IL6; urea nitrogen and calcium-phosphorus product showed mild decreases during the washout period	(Gholami et al., 2023)

FMT, fecal microbiota transplantation; TCM, traditional Chinese medicine; HD, hemodialysis; PD, peritoneal dialysis; RCT, randomized controlled trial; NA, not assessed. UTs detected include indoxyl sulfate (IS), p-cresyl sulfate (pCS), and trimethylamine N-oxide (TMAO). SCFAs, short-chain fatty acids; eGFR, estimated glomerular filtration rate; Scr, serum creatinine; BUN, blood urea nitrogen. Preclinical entries refer to adenine- or cisplatin-induced CKD mouse models. All the data were extracted from the cited original research literature.

### 5.1 FMT

FMT restores intestinal microbial functionality by transplanting viable microbial communities from healthy donor feces into the recipient’s gastrointestinal tract (Gholami et al., 2024; Kim et al., 2024). Its application in constipation and CKD remains investigational. The standard FMT procedure involves three key steps: selecting suitable donors and donor fecal microbiota and administering them via conventional routes such as enema, colonoscopy, nasoduodenal or jejunal infusion, or capsule FMT (cFMT) (Zhang et al., 2018).

FMT has been shown to improve symptoms of slow-transit constipation in non-CKD patients and alter the diversity of fecal α/β, involving changes in the gut microbiota, such as that of *Prevotella*, *Bacteroides*, *Roseburia*, and *Blautia* (Xie et al., 2021; Hou et al., 2025). Specific clinical data on FMT for patients with ESRD/dialysis complicated by constipation are very limited, and existing evidence primarily comes from nondialysis chronic kidney disease populations. An RCT including 28 patients with stage 2–4 chronic kidney disease (with diabetes or hypertension) revealed that oral FMT capsules resulted in more stable serum creatinine and blood urea nitrogen levels and a slower decline in the eGFR than placebo did (Arteaga-Muller et al., 2024). An observational study of 22 chronic kidney disease patients revealed that 24-hour urinary protein levels significantly decreased 6 months after FMT. The serum creatinine concentration decreased from 108.333 μmol/L to 102.933 μmol/L, the eGFR increased from 77.530 mL/min/1.73 m^2^ to 83.952 mL/min/1.73 m^2^, and gastrointestinal symptoms improved in 72.7% of patients (Guo et al., 2025).

Preclinical studies suggest that FMT can reshape the structure of the gut microbiota, thereby potentially improving intestinal motility, repairing intestinal barrier integrity, and reducing the production of UTs. These mechanisms provide a biological rationale for applying FMT to patients with constipation associated with ESRD; however, this conclusion has not yet been validated in large-scale, multicenter ESRD patients. For dialysis patients with central venous catheters and compromised immune function, FMT may increase the risk of bacteremia and systemic infections. Therefore, strict pathogen screening and antibiotic resistance gene testing of donors are crucial. Additionally, the optimal route of administration, dosage, and frequency of FMT for ESRD patients remain unclear. Owing to individual variations in baseline gut microbiota dysbiosis, treatment responses may be highly heterogeneous, and systematic data on long-term safety are lacking. Thus, clinical application of FMT requires caution.

### 5.2 Traditional Chinese medicine and TCM decoctions

TCM has unique advantages in treating diseases because of its use of multicomponent, multitarget, and multipathway approaches. TCM and its formulations also show good efficacy in treating constipation. In recent years, numerous studies have confirmed that various TCM herbs and their active components can manage constipation by modulating the gut microbiota structure, promoting SCFA-induced bacterial proliferation, repairing the intestinal barrier, and improving intestinal motility (Wang et al., 2024).

*Cassia fistula* syrup (CFS) contains Cassia fistula, an important traditional medicinal plant that has been proven to have antibacterial and constipation-relieving effects (Mwangi et al., 2021). Additionally, other species of the same genus, such as *C. angustifolia*, *C. fistula*, and *C. alata*, exhibit laxative-like effects, primarily attributed to anthraquinone derivatives (Aslam, 2019). A randomized controlled clinical trial involving 66 CKD patients treated with CFS or lactulose revealed that CFS significantly improved constipation in CKD patients, with effects superior to those of lactulose (Jahanian et al., 2025).

Tiaopi Xiezhuo decoction (TXD) is a traditional Chinese herbal formula composed of *Rheum palmatum* L. (Polygonaceae), *Coptis chinensis* Franch. (Ranunculaceae), and *Zingiber officinale* Roscoe (Zingiberaceae) at a 1:1:1 ratio. *R. palmatum* and *C. chinensis* in the TXD have been confirmed to regulate the gut microbiota (Ji et al., 2020; Lyu et al., 2021). In a study of 29 PD patients treated with TXD, a 3-month TXD intervention alleviated constipation by increasing the diversity of the gut microbiota and increasing the abundance of species such as *Paraprevotella clara*, *Phascolarctobacterium succinatutens* and *Prevotella copri* (Peng et al., 2023).

Bushen Qudu Formula is an oral traditional Chinese medicine compound formula composed of processed Polygonum multiflorum, *Cornus officinalis*, Phellodendron amurense, Dragon’s blood, and *Polygonum cuspidatum* rhizome. In a randomized controlled trial involving 139 patients with maintenance hemodialysis-associated pruritus, patients who orally received the Bushen Qudu Formula showed significantly better improvement in constipation scores than did the cetirizine treatment group did. Additionally, serum blood urea nitrogen, C-reactive protein, and potassium levels were significantly reduced. Bushen Qudu Formula may help alleviate constipation symptoms in hemodialysis patients and potentially reduce microinflammatory status and azotemia (Zhang et al., 2026).

TCMs frequently exhibit nonuniform compositions and inconsistent batch quality, which complicates the determination of dose–response relationships. Potential interactions between TCM and commonly prescribed medications for ESRD, including antihypertensives, phosphate binders, and immunosuppressants, have not been systematically evaluated. Certain TCM components may contain nephrotoxic substances or accumulate in individuals with impaired renal function. Specialized kidney safety studies specifically targeting end-stage renal disease populations are lacking.

### 5.3 Probiotics, prebiotics, synbiotics, postbiotics

Numerous clinical trials and experimental studies have shown that the administration of prebiotics, probiotics, and synbiotics can reduce the levels of uremic IS, PCS, and inflammatory mediators (Ramezani et al., 2016; Rossi et al., 2016) and alleviate the destruction of tight junctions in colonic epithelial cells, thereby significantly improving endotoxemia, blood urea nitrogen levels, and quality of life (Vaziri et al., 2014).

Probiotics refer to microorganisms that confer health benefits to the host, including live *Bifidobacterium*, *Lactobacillus* and *Streptococcus*, which can alter the gut microbiota (Leelahavanichkul et al., 2025). After a 4-week washout period, patients with nondialysis CKD (stages 3–5, GFR 10–60 mL/min/1.73 m^2^) received oral L34-encapsulated powder containing 3.5×10^9^ CFU at a fixed morning time for 4 weeks. Probiotic treatment altered the gut microbiota, reducing the levels of UTs (PCS and IS) and inflammatory cytokines. Cell experiments confirmed that L34 attenuated the damage caused by UTs to intestinal cells, macrophages, and neutrophils (Leelahavanichkul et al., 2025). In a multicenter clinical study, patients with CKD complicated by constipation were administered an investigational drug (a tablet containing *Bifidobacterium* strain G9-1) at a dosage of 2 tablets per administration three times daily for a duration of 12 weeks. By the sixth week, there was a significant decrease in urinary protein levels and an increase in bowel movement frequency. At the 12-week mark, the proportion of patients with normalized constipated stools (44.8%; 13 cases) was significantly greater than that at baseline (Ikeda et al., 2026). The use of probiotics significantly alters the composition of the gut microbiota and effectively relieves constipation.

Prebiotics are indigestible food components, including dietary fiber, oligosaccharides, polysaccharides, and resistant starch, that stimulate the growth or activity of SCFA-producing bacteria in the colon and reduce the production of UTs (Hutkins et al., 2016). In a clinical trial, 15 HD patients received partially hydrolyzed guar gum as a prebiotic intervention twice daily for 4 weeks. The results revealed improved stool consistency; reduced constipation severity; increased abundance of *Bifidobacterium*, Bacteroidota and *Lactobacillus*; and elevated short-chain fatty acid concentrations (Miyoshi et al., 2020).

Synbiotics refer to a combination of probiotics and prebiotics that increase the survival and activity of beneficial gut microbes (Rossi et al., 2016). In a randomized controlled trial, 60 HD patients were divided into a synbiotic group and a control group. The synbiotic intervention consisted of a compound preparation containing *Lactobacillus*, *Bifidobacterium* and fructooligosaccharides. After 60 days of continuous treatment, the synbiotic group showed significant improvement in symptoms of constipation and improved quality of life (Lydia et al., 2022). Additionally, in patients with stage IIIb-IV CKD treated with a novel synbiotic formulation, compared with patients in the placebo group, those in the synbiotic group exhibited significantly lower IS levels and reduced small intestinal permeability (Cosola et al., 2021).

Postbiotics refer to nonviable bacterial products or microorganisms, including probiotic metabolic byproducts such as SCFAs, bacterial lysates and supernatants, extracellular polysaccharides, bioactive peptides, organic acids, etc. Due to the absence of live microorganisms, their risk is lower than that of probiotics (Żółkiewicz et al., 2020). Geng et al. treated CKD mice with the inactivated *Lactobacillus plantarum* strain MA2 (IMA2), resulting in reduced serum UTs levels, increased claudin-1 expression, and decreased levels of LPS, TLR4, and IL-1β in the kidneys. Additionally, it increased the abundance of lactobacilli and reduced the abundance of proteolytic bacteria, thereby regulating the structure of the gut microbiota (Geng et al., 2025). Gholami et al. reported that the supernatant of *Lactobacillus fermentum* significantly reduced blood urea nitrogen and creatinine levels, as well as ROS formation and lipid peroxidation, thereby improving renal function (Gholami et al., 2023). Clinical data on postbiotics in human ESRD patients are currently unavailable.

Overall, interventions targeting the gut microbiota have shown promising initial signals for improving constipation and modulating the microbial composition in CKD/ESRD patients, but the evidence remains immature. First, most clinical trials are small, single-center, and short-term, with significant heterogeneity in intervention formulations, dosages, and outcome measures. Second, studies often rely on surrogate end points (such as serum toxin levels and changes in microbial composition) rather than hard clinical end points (such as a decline in renal function, hospitalization, or mortality). Third, evidence specific to dialysis/ESRD populations is extremely limited, with many findings extrapolated from nondialysis CKD or non-CKD constipation cohorts. Fourth, the safety of most interventions has not been systematically evaluated in the vulnerable ESRD population. Larger, multicenter, long-term RCTs with standardized endpoints, including optimal strain selection, dosage, and duration of treatment, are needed to establish their clinical benefits and safety.

## 6 Summary and future perspectives

ESRD patients may experience constipation induced or exacerbated by various factors, such as side effects of medications (Cha et al., 2023), comorbid conditions (Xi et al., 2023), and dietary restrictions (Kosmadakis et al., 2019; Barberio et al., 2021; Wan et al., 2023), leading to reduced quality of life scores in constipated patients. In summary, this review systematically integrates the bidirectional association between gut microbiota dysbiosis and constipation in ESRD patients and proposes a bidirectional “microbiota–gut–kidney” vicious cycle centered on constipation. ESRD and its renal replacement therapies drive gut microbiota dysbiosis via multiple pathways, marked by reduced short-chain fatty acid-producing bacteria, enrichment of proteolytic and urease-positive bacteria, and decreased microbial diversity. Conversely, microbiota dysbiosis exacerbates constipation and accelerates renal function deterioration through three interconnected mechanisms: gastrointestinal motility dysfunction, intestinal barrier disruption, and amplification of gut–kidney uremic toxins, resulting in the formation of a self-reinforcing pathological cycle. Current interventions targeting the microbiota, including FMT, TCM and compound preparations, probiotics, prebiotics, synbiotics, and postbiotics, have shown promising initial efficacy in relieving constipation symptoms, regulating microbial composition, and reducing circulating uremic toxin levels. However, the evidence remains immature, particularly regarding high-quality clinical trials and systematic safety data in dialysis populations. We challenge the traditional view of constipation as a secondary ESRD complication, offering a novel pathophysiological perspective for mechanism-based interventions to delay disease progression and improve patient outcomes.

### 6.1 Future perspectives

Current research is largely limited to the analysis of changes in microbial composition and metabolite associations, and several critical translational gaps remain to be addressed. First, standardized and validated diagnostic criteria for constipation (e.g., Rome IV criteria) should be adopted, along with hard clinical outcome measures such as constipation resolution, quality of life, incidence of hyperkalemia, hospitalization rate, infection rate, dialysis adequacy, and decline in residual renal function. Additionally, key clinical covariates should be consistently reported to reduce methodological heterogeneity across studies. Second, multicenter prospective cohort studies with longitudinal sampling before and after dialysis and throughout the treatment period should be conducted to establish the temporal relationships among gut dysbiosis, the onset of constipation, and the decline in renal function, thereby strengthening causal inference. Third, confounding factors such as dietary fiber restriction, polypharmacy, and physical inactivity need to be systematically controlled to quantify the independent contribution of constipation to microbial alterations and the progression of end-stage renal disease. Fourth, artificial intelligence and big data analytics should be used to develop risk prediction models for gut dysbiosis and constipation in the end-stage renal disease population, enabling early identification and preventive intervention for high-risk patients. Fifth, given the lack of specificity in probiotic strain selection, personalized intervention strategies should be developed on the basis of an individual’s baseline microbiota profile, constipation severity, and renal function. By optimizing strain selection, formulation, and treatment regimens, therapeutic consistency can be improved. Moreover, integrating TCM with gut microbiota-targeted therapy, exploring the mechanisms by which TCM modulates the gut microbiota, and establishing a “targeted TCM” comprehensive treatment model may enhance therapeutic efficacy.

Additionally, the specific regulatory pathways of key microbial taxa and their metabolites at the mechanistic level remain unclear. Future work should integrate metagenomics, metabolomics, and transcriptomics to move beyond taxonomic correlations and identify core functional microbial pathways and key metabolic targets mediating the link between constipation and renal injury. Particular emphasis should be placed on elucidating how microbial metabolites regulate intestinal smooth muscle function, intestinal barrier integrity, and renal fibrosis through specific molecular mechanisms, including enteric neurotransmitter modulation, barrier repair, and toxin-mediated fibrosis. Functional validation using intestinal organoids, germ-free mice, and colonization animal models should be performed to confirm the causal roles of specific microbial taxa or metabolites.

Furthermore, at the mechanistic level, the specific regulatory pathways of key microbial taxa and their metabolites remain unclear. Future studies should integrate metagenomics, metabolomics, and transcriptomics to move beyond taxonomic correlations and identify the core functional microbial pathways and key metabolic targets that mediate the link between constipation and kidney injury. In particular, efforts should focus on elucidating how microbial metabolites regulate intestinal smooth muscle function, intestinal barrier integrity, and renal fibrosis through specific molecular mechanisms, including enteric neurotransmitter modulation, barrier repair, and toxin-mediated fibrosis. Functional validation using intestinal organoids, germ-free mice, and colonized animal models should be performed to confirm the causal roles of specific microbial taxa or metabolites.

In advancing the above clinical translation, safety considerations are particularly important for end-stage renal disease and dialysis patients and must be given high priority. Future research should consider the immunocompromised status and frequent use of central venous catheters in this population and systematically evaluate the safety profiles of all microbiota-directed interventions in dialysis patients. Key safety concerns include the transmission of pathogens and antimicrobial resistance genes in fecal microbiota transplantation, the risk of bacteremia caused by live probiotic strains, and the potential nephrotoxicity and herb–drug interactions of herbal preparations. Standardized operating procedures and safety monitoring systems for microbiota-targeted interventions specific to the end-stage renal disease population should be established to fill the current gaps in clinical practice guidelines for dialysis patients. Key bottlenecks in translational research must be identified and resolved to facilitate the conversion of basic mechanistic findings into clinically applicable diagnostic and therapeutic tools.

## 7 Limitations

Although this review comprehensively summarizes the role of gut microbiota dysbiosis in the “gut–kidney” axis, its regulatory mechanisms in ESRD, and its pathogenic association with constipation, several limitations persist. First, although quantitative data on UTs and SCFAs are provided as supplementary information, disparities in detection indicators, sampling methods, and study populations across original investigations preclude a unified, standardized comparative analysis between healthy individuals and ESRD patients. Furthermore, variations in dialysis modalities (HD vs. PD), residual renal function, comorbidity profiles, and geographic backgrounds complicate the derivation of a universal microbial signature linked to ESRD-related constipation. Second, the review did not systematically compare differences in dietary patterns and drug interventions between healthy control groups and ESRD patients, as dietary status is a key factor that regulates gut microbiota homeostasis. Third, the effects of routine ESRD treatment medications on the human gut microbiota composition were neither analyzed nor discussed. Both dietary interventions and pharmacological interventions may influence gut microbiota dysbiosis and related metabolite profiles, thereby indirectly affecting the outcomes of correlation analyses. Fourth, most available human studies are observational, limiting the ability to establish temporal sequences or causal relationships between gut microbiota dysbiosis, constipation, and ESRD progression; mechanistic evidence is derived primarily from animal models or *in vitro* experiments and remains at the preclinical research stage. Future studies should aim to standardize research conditions, rigorously control dietary variables, and stratify patient cohorts by medication regimens to further validate the relationship between alterations in the gut microbiota and ESRD progression.
